# Prevalence of angina in patients with systemic lupus erythematosus

**DOI:** 10.1186/ar3996

**Published:** 2012-09-27

**Authors:** ML Ishimori, NJ Gal, A Rogatko, DS Berman, A Wilson, DJ Wallace, NB Merz, MH Weisman

**Affiliations:** 1 Cedars-Sinai Medical Center, Los Angeles, CA, USA; 2 Samuel Oschin Comprehensive Cancer Institute, Cedars-Sinai Medical Center, Los Angeles, CA, USA; 3 Women's Heart Center, Los Angeles, CA, USA

## Background

The study of coronary artery disease (CAD) in systemic lupus erythematosus (SLE) has become increasingly important with evidence indicating a relationship between cardiovascular disease outcome and chronic inflammatory conditions [1]. Since anginal chest pain (CP) is a frequent clinical symptom in patients with CAD, it is important to estimate its frequency among SLE patients. The Rose angina questionnaire and the Diamond-Forrester index are widely used tools to assess angina in the general population but have not previously been used to measure angina prevalence in SLE [2,3]. We conducted a pilot study on the prevalence of self-reported CP in patients with SLE.

## Methods

Informed consent was obtained from consecutive adult SLE patients presenting to a rheumatology practice between January 2010 and April 2010. All patients were assessed by American College of Rheumatology criteria for SLE and confirmed to have SLE by the rheumatologist and by chart review. Two self-administered questionnaires were completed on a single visit. The Rose angina questionnaire assessed whether patients have ever had CP or angina and the Diamond-Forrester index ascertained whether patients were experiencing current angina. For data management and statistical analysis, SAS 9.1 and STATA 10 were employed. The confidence intervals represent Wald confidence intervals of the proportion ± 1.96× standard error truncated to zero or one in cases where the limits have been outside that range. Atypical or typical angina by the Diamond index was considered angina.

## Results

A total of 150 SLE subjects were enrolled (94% female, mean age 43 ± 13 years, mean disease duration 12 ± 9 years). Ninety-six subjects (65.8%; 95% CI = 58.0 to 73.5) indicated they had ever experienced CP. A history of angina by Rose angina questionnaire was reported in 18 patients (12.3%; 95% CI = 6.9 to 17.7). Thirty-one (21.2%; 95% CI = 14.5 to 27.9) patients indicated they were experiencing current CP and were administered the Diamond index; 12 (8.2%; 95% CI = 3.7 12.7) subjects had current angina by Diamond index, including two patients (1.4%; 95% CI = 0 to 3.3) with typical angina. No relationship was found between age or disease duration and scores on either questionnaire.

## Conclusion

Our data indicate a 12.3% prevalence of ever having had angina, and an 8.2% prevalence of current angina. A recent meta-analysis of worldwide responses to the Rose angina questionnaire found the population weighted mean of angina prevalence to be 6.7% in women and 5.7% in men [4]. It appears that the prevalence of angina history in SLE patients is approximately twice that in the general population.
